# Adaptation options for wheat in Europe will be limited by increased adverse weather events under climate change

**DOI:** 10.1098/rsif.2015.0721

**Published:** 2015-11-06

**Authors:** Miroslav Trnka, Petr Hlavinka, Mikhail A. Semenov

**Affiliations:** 1 Global Change Research Centre AS CR v.v.i., Bělidla 986/4b, Brno 60300, Czech Republic; 2 Institute of Agrosystems and Bioclimatology, Mendel University in Brno, Zemědělská 1, Brno 61300, Czech Republic; 3 Department of Computational and Systems Biology, Rothamsted Research, Harpenden AL5 2JQ, UK

**Keywords:** climate change, extreme events, food security, winter wheat

## Abstract

Ways of increasing the production of wheat, the most widely grown cereal crop, will need to be found to meet the increasing demand caused by human population growth in the coming decades. This increase must occur despite the decrease in yield gains now being reported in some regions, increased price volatility and the expected increase in the frequency of adverse weather events that can reduce yields. However, if and how the frequency of adverse weather events will change over Europe, the most important wheat-growing area, has not yet been analysed. Here, we show that the accumulated probability of 11 adverse weather events with the potential to significantly reduce yield will increase markedly across all of Europe. We found that by the end of the century, the exposure of the key European wheat-growing areas, where most wheat production is currently concentrated, may increase more than twofold. However, if we consider the entire arable land area of Europe, a greater than threefold increase in risk was predicted. Therefore, shifting wheat production to new producing regions to reduce the risk might not be possible as the risk of adverse events beyond the key wheat-growing areas increases even more. Furthermore, we found a marked increase in wheat exposure to high temperatures, severe droughts and field inaccessibility compared with other types of adverse events. Our results also showed the limitations of some of the presently debated adaptation options and demonstrated the need for development of region-specific strategies. Other regions of the world could be affected by adverse weather events in the future in a way different from that considered here for Europe. This observation emphasizes the importance of conducting similar analyses for other major wheat regions.

## 1. Introduction

The 70% increase in food production by 2050 required to feed a population over 9 billion [1] puts pressure on the production of sufficient amount of high-quality food in a sustainable way [2]. At the same time, ongoing climate change with warming trends across the globe has resulted in increased climate variability and extremes [3–6], although high uncertainty remains in the relationship between global warming and variability [7]. There is serious concern about deteriorating food quality [8] and abnormally high volatility in food prices arising from their close connection to the price of crude oil [2]. Challinor *et al.* [9] estimated that, for 2°C of local warming without adaptation, losses in aggregate production could be expected for wheat, rice and maize in both temperate and tropical regions, while using available adaptation options may lead to a 7–15% yield increase. Such yield increases would fall short of the 70% required. The majority of existing studies [9] do not fully consider the impacts of adverse weather events (i.e. conditions capable of causing severe yield reductions). While there has been an effort to increase the reliability of future yield predictions through the use of ensembles of crop models [10], these models still do not incorporate the effects of most adverse weather events. The same is true for design of wheat ideotypes (and breeding priorities) for target environments [11].

**Figure 1.**
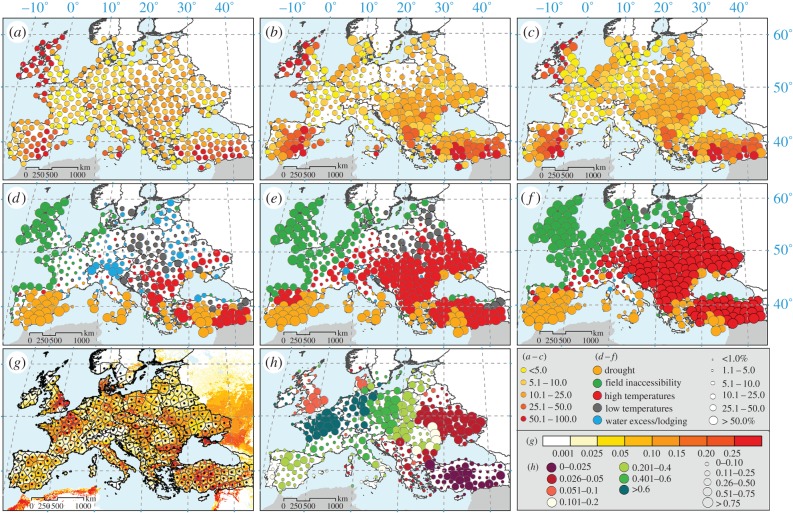
Combined probability of a single adverse event over (*a*) the baseline, (*b*) GISS-RCP8.5 and (*c*) HadGEM-RCP8.5 scenarios with the size of the circle corresponding to the relative change compared to the baseline. (*d*) The dominant type of adverse event for the baseline, (*e*) for GISS-RCP8.5 and (*f*) for HadGEM-RCP8.5 with the size of the circle corresponding to the event frequency. (*g*) Proportion of wheat area in each grid (colour) in Europe based on Monfreda *et al.* [14] with the locations of 379 sites used in the study (thin lines are Thiesen polygons). (*h*) Colour-coding corresponds to the share of European wheat production per polygon, and the size of the circle corresponds to the proportion of the European wheat area represented by the polygon. Baseline (1981–2010) and climate scenarios (2081–2100).

In this study, we seek to answer the following research question: ‘To what extent will climate change alter the probability of those adverse weather events that can be detrimental to wheat production?' The study is focused on wheat as the most widely grown crop in terms of harvested area [12]. Wheat provides approximately 20% of total human calorie consumption [13]. World trade in wheat is greater than for all other crops combined and, in terms of the total production tonnages used for food, it is currently second to rice as the main human food crop and is the leading source of vegetable protein in human food [8]. The area covered by the study (figure 1) represents almost one-third of global wheat production and exports [13]. Production (depending on the region) is being affected by high temperatures, occurrences of drought, late spring frosts and severe winter frosts associated with inadequate snow cover, lodging, waterlogging and field accessibility during key field operations [15]. This study employs recently developed methodology applicable for the assessment of combined probability of multiple adverse events affecting wheat production [14] with local-scale climate scenarios based on two selected Global Circulation Models (GCMs) from the most up-to-date CMIP5 [16] multi-model ensemble. Realizing the critical importance of European growing areas [15], we also tested the potential benefits of some of the adaptation strategies.

## 2. Results

### 2.1. Change in the adverse event probability and dominant type

**Figure 2.**
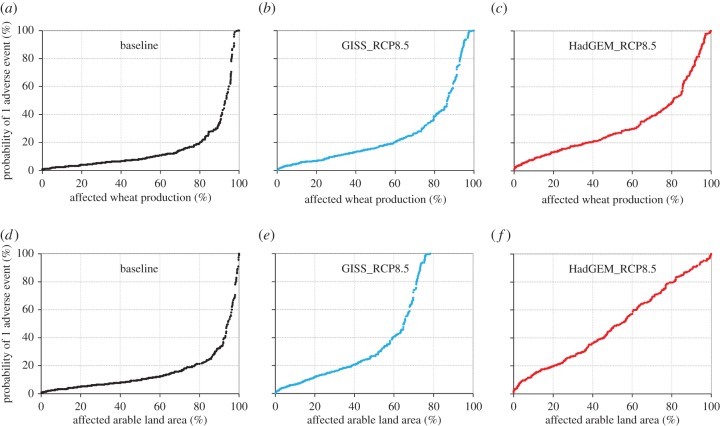
Cumulative probability of the occurrence of a single adverse event during wheat-growing season under baseline and projected climate scenarios as a function of (*a–c*) the affected wheat production, and (*d–f*) the arable land area. (*a,d*) represent the baseline period (1981–2010) and (*b,c,e,f*) climate scenarios (2081–2100) for low-sensitivity GISS (*b,e*) and high-sensitivity HadGEM (*c,f*) climate models for RCP8.5. A medium-ripening cultivar was used in the simulations.

**Figure 3.**
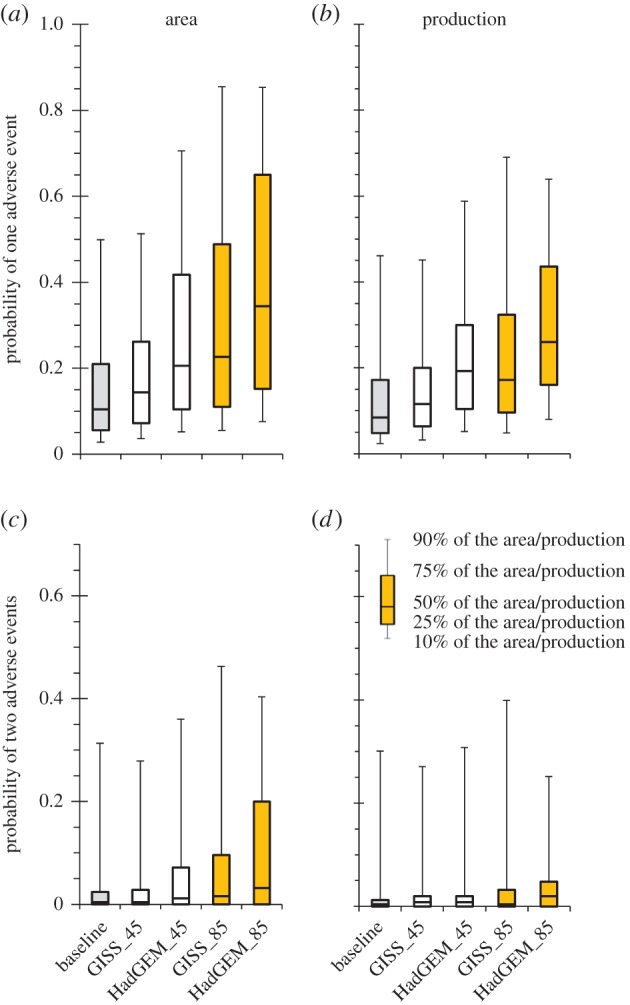
Boxplots of probability of one (*a,b*) and two (*c,d*) adverse events for the wheat area (*a,c*) and wheat production (*b,d*) for the baseline (grey), RCP4.5 (white) and RCP8.5 (orange) climate scenarios from the GISS and HadGEM climate models. Each boxplot shows the mean adverse event probability for the top 10, 25, 50, 75 and 90% grids according to their wheat area (*a,c*) or their wheat production (*b,d*). Baseline (1981–2010) and climate scenarios (2081–2100).

Under the present climate, the probability of a single adverse event is lower than 20% (i.e. once every 5 years) over the wheat-growing area that delivers 80% of the wheat in Europe (figure 2*a,d*). The core areas producing more than one-half of all European wheat are faced with some type of adverse event at least once every 10 years (figure 2*a*). Under both RCP4.5 and RCP8.5 scenarios, the probability of a single adverse event is predicted to increase considerably (figures 2 and 3). Under the HadGEM-RCP8.5 climate scenario, by 2090 only 10% of European wheat production would be affected by a single adverse event less than once every 10 years, while one-half of the arable land area of Europe would be affected at least once every 2 years (figure 2*f*). There is a significant difference in the probabilities of a single adverse event between climate scenarios based on RCP4.5 and RCP8.5, with the latter showing a much greater increase in risk (figure 3). There are also considerable differences (figures 2 and 3) in the probabilities of a single adverse event between climate scenarios based on the low-climate-sensitivity model (GISS) and the high-climate-sensitivity model (HadGEM). However, even a relatively ‘favourable’ climate scenario based on projections from GISS for RCP4.5 indicates a notably higher overall adverse event frequency. Figure 3*a,b* shows that the key areas where the majority of European wheat is produced today are less vulnerable to changes in the frequency of adverse weather events compared with the entire arable land area. At present, therefore, most European wheat is grown in areas with a lower risk of adverse events relative to European arable land as a whole. Despite this, the exposure of the major wheat-producing areas to adverse events is predicted to increase more than twofold for the RCP8.5 and HadGEM model compared with a threefold increase over the entire available area of Europe's arable land.

Field inaccessibility under current conditions is indicated to be the most frequent limitation for wheat production over much of the northwestern coastal area of the continent and the UK. Drought is estimated to be the major limiting factor for wheat growth in parts of the Mediterranean, with heat stress affecting parts of southeastern Europe and Turkey. According to our estimates, water excess and lodging risk, together with the occurrence of low temperatures, are the major concerns in central and northeastern Europe (figure 1*d*). Future climatic conditions by the end of the century (2081–2100) based on the RCP8.5 emission scenario would lead to not only a sharp increase in the probability of these events, as discussed, but also more distinct regionalization of the dominant adverse events, with the northwestern coast being even more affected by field inaccessibility and almost all of southeastern and central Europe being affected by a significant increase in exposure to high temperatures (figure 1*e,f*). Drought would remain dominant on the Iberian Peninsula and in parts of Turkey. The major difference between the HadGEM and GISS scenarios (figure 1*f,e*) is that the former would bring a more significant increase in adverse weather events for the most productive areas indicated in figure 2.

### 2.2. Implications for adaptation strategies

One of the adaptation strategies for reducing the risk of adverse weather events is stress avoidance through shifts in either time or space. Avoidance in time could be achieved by using early ripening cultivars to escape heat or drought stress or moving the focus of production elsewhere. However, electronic supplementary material, appendix figure S1 shows that this avoidance strategy would lead to a lower sum of effective global radiation (EfGr—see the electronic supplementary material, appendix for a more detailed description) intercepted by the crop, decreasing yield potential (unless the radiation-use efficiency of the crop were significantly improved). Under future climate scenarios, not only was the EfGr reduced by shifting the wheat-growing season to a period of shorter days, but droughts also became more frequent and prolonged, especially in south and southeast Europe (electronic supplementary material, appendix figure S1).

Avoidance in space could be achieved by shifting wheat production to new growing areas which could potentially be less endangered by the projected increase in adverse event frequency. While regions with very small or even no change in the adverse event frequency can be found (figure 3*a–c*), overall the most important wheat-producing areas will be faced with an up to twofold increase for the RCP8.5 and HadGEM models. However, the increase in the adverse event frequency over the entire available area of arable land in Europe is more than threefold compared with the present conditions. This indicates that shifting wheat production to areas not currently used for wheat would lead to an even higher probability of adverse events (figure 1). Therefore, growing wheat predominantly in the present growing area (and adaptation to the increase in the frequency of adverse events) seems to be the most likely scenario.

We also assessed the strategy that would be focused on maximizing the EfGr by maintaining the length of the growing period, using late-maturing cultivars, and we asked whether this strategy would lead to an increased exposure to adverse weather events (figure 3). Obviously, different-maturing cultivars are used in different regions based on multiple factors and operational requirements. In electronic supplementary material, appendix figure S2*g*–*i*, we selected, for each growing season, the cultivar that had the lowest risk of adverse events without significantly reducing the total EfGr. The use of early maturing cultivars in the Mediterranean and southeastern Europe under the model's future climate (i.e. 2081–2100) resulted in a significant reduction of adverse events compared to the medium cultivars, while the late-maturing cultivars performed the best at some sites. However, as the electronic supplementary material, appendix figure S2*d*–*f* shows, the stress avoidance strategy only partly succeeded in reducing the exposure of the wheat crop to the adverse events, and the overall risk would still be far greater than under present conditions. Opting for this strategy would also mean a decrease of the EfGr available for wheat growing in what would then be the most productive regions of Europe, resulting in a decrease in yield potential (electronic supplementary material, appendix figure S1*h*).

## 3. Discussion

Existing studies estimating the effect of climate change on production (e.g. [9,17]) rely either on the set of empirical models or process-based crop models that are not primarily optimized to recognize the impacts of most of adverse events that were considered here. This is the case for the former class of models because of their reliance on monthly or seasonal data; this is also the case for the latter class because the model algorithms do not account fully (or at all) for the effects of these adverse events, which in reality can cause major yield decreases. While there has been great effort focused on crop model improvement (e.g. [10]), there has to date not been a concerted effort focusing on the adverse weather events relevant to wheat as such. Even now, targeting genotypes that provide good matches to environments still relies on monthly or even seasonal climatological parameters (e.g. [11]) and does not reflect the baseline frequency of adverse events. In those cases where research focuses on extreme/adverse weather, there seems to be a bias towards certain types of these events, which are then addressed in detail without considering other coexisting potential threats. In recent years, substantial research efforts have focused on the effect of drought and especially of heat stress (e.g. [4,18,19]). However, it is well known that wheat production is affected by not only the frequency of days with high temperatures but also the occurrence of late frosts and severe frosts without adequate snow cover or by overly wet and cool weather, which enhances the occurrence of diseases, contributes to lodging and makes crop management more difficult. Many efforts have also been (rightly) focused on those regions of the world where the understanding of the climate–yield relationship is less advanced, e.g. West, Central or East Africa [17]. However, the eventual changes in the production patterns in areas in the key exporting countries/regions, as performed in this case or by Moore & Lobell [20], are as important for the stability of global food prices as production in those countries that are key producers but use most of their production domestically (e.g. China or India). The cited study concluded that long-term temperature and precipitation trends since 1989 have reduced continent-wide wheat yields by 2.5%, with a high level of heterogeneity across the continent and the highest impact observed around the Mediterranean. While the climate trends according to this study can account for only 10% of the stagnation in European wheat yields, it should be remembered that observed climate trends are expected to accelerate significantly, particularly if RCP 8.5 is considered. Our results showed that these changes would lead to a several-fold increase in the risks of single and multiple adverse events occurring within one wheat-growing season. This also increases the risk that compared with the baseline, events capable of significantly reducing wheat yields will occur across a wider part of the European wheat-growing area within the same season. When the shift of the wheat production to other parts of Europe as a coping strategy was explored authors did not consider other factors influencing such a shift, e.g. local demand, farmer skill, suitable soils, production infrastructure or need to displace currently produced crops. However in the case of changing climate conditions and adverse event risk it is fair to assume that shift of the wheat area production will be considered as one of the options.

Negative impacts of increased risks of adverse weather events would likely not be limited only to wheat crops. The growing seasons, sensitive periods and magnitude of impacts of many important crops overlap with those of wheat [21]. Therefore, it is likely that the productivity of these other crops will also be affected by increased adverse weather events. Olesen *et al.* [22] showed that spring crops tend to be even more sensitive to some of the evaluated events (e.g. drought) than are winter crops. While spring crops would apparently not suffer directly from low winter temperatures their sowing would be affected by the increased water stagnation projected for some of the regions. Adaptation to some of these adverse events would be difficult and costly.

As Trnka *et al.* [15] have shown, the severity and frequency of some of the adverse events (e.g. drought stress) would, in general, be higher on soils with a lower water-holding capacity, both under baseline and future climate scenarios (i.e. 2081–2100). This finding was confirmed also in our study when we used free-draining soil with a water-holding capacity of 270 mm and compared it with a ‘light’ soil with its water-holding capacity set at 150 mm. The water-holding properties of the selected soil used in the study are very good in comparison with the majority of the arable land. If we select actual soils (with water-holding capacity in many cases below 270 mm), then the probability of adverse events as defined in the study will mostly increase. By contrast, the study did not consider the influence of the high underground water table that could be found at some of the key wheat-producing areas in Europe [23] and would be capable of mitigating the impact of some adverse events (especially of the drought stress), but, equally, could prolong periods of water stagnation if the water table itself remained high. Our study also did not consider irrigation, as the absolute majority of Europe wheat production is rainfed. While the use of irrigation would decrease the overall exposure of wheat crop to some adverse weather events and would eventually allow a longer growing season it would be limited by the existing water scarcity in some areas and by the existing legal requirements (e.g. [24]).

By using climate scenarios based on low- and high-climate-sensitivity GCMs from the CMIP5 ensemble, we estimated the range of responses which, in principle, should be comparable with the range based on the whole CMIP5 ensemble. Under future climate scenarios, nearly every site is at risk of different multiple events depending on the scenario and the region. Therefore, the target traits for wheat improvement and strategies focused on improvement of the field conditions vary according to the region and the magnitude of climate change. Failing to address these challenges by appropriate adaptation measures could potentially lead to a substantial reduction of European wheat production in the future. Other key wheat-producing regions of the world could be affected differently by changes in adverse weather events in the future, including other dominating types of adverse events or different magnitudes of these changes. Therefore, similar analyses for other major wheat-growing regions should be performed in addition to impact assessments based on crop growth models.

## 4. Methods

The paper focuses on events that could be considered ‘adverse’, i.e. conditions that are detrimental to winter wheat yield. The specific thresholds are described in the electronic supplementary material, appendix table S1. We prefer the term ‘adverse’ rather than ‘extreme’, as the latter term is usually defined by the frequency of occurrence at the given site/region.

### 4.1. Study area and climate data

The simulation of adverse weather events for wheat was performed for 379 European sites that represent the study domain (figure 1). In total, 36 European countries are represented by the study, covering the current European wheat-producing regions with the exception of Russia (figure 1*g*). Two GCMs from the CMIP5 ensemble were used with low, GISS-E2-R-CC (GISS), and high, HadGEM2-ES (HadGEM), climate sensitivity (electronic supplementary material, appendix figure S3). Two representative concentration pathway scenarios, RCP4.5 and RCP8.5, were considered in the construction of local-scale climate scenarios. Climate projections from GCMs were downscaled to the local-scale daily weather by the LARS-WG 6.0 weather generator using the ELPIS dataset of site-specific parameters across Europe [25,26]. For each site and for each combination of GCMs and RCPs, we generated 300 years of daily site-specific weather, representing the baseline scenario corresponding to 1981–2010, and 300 years for the future climate scenario corresponding to 2081–2100. In each simulation, the first 50 years were used to initiate the calculation, and the remaining 250 years of data were retained for the subsequent analyses.

### 4.2. Agroclimate modelling

For each site, we used three types of cultivars according to the maturity date and two levels of photoperiod sensitivity as described by Trnka *et al.* [15]. The sowing, anthesis and maturity dates for the baseline conditions were estimated using AgriClim software, with the mean dates presented in the electronic supplementary material, appendix figure S4. It is assumed that cultivars represent winter wheat in all locations except those where temperature constrains vernalization. At these locations, we assumed that winter-sown spring wheat cultivars are used. For the entire study, autumn sowing dates were preferred to keep the sowing within the same season for all locations and facilitate comparisons among them. The duration of phenological phases was calculated according to Olesen *et al.* [27] using accumulated degree days (°Cd) above the base temperature combined with the day-length response for the period from emergence to anthesis as used by Trnka *et al.* [15]. The detailed settings are listed in the electronic supplementary material, appendix table S2. The sowing dates were determined automatically as the first day after the mean air temperature dropped below 13°C for more than five subsequent days with the soil moisture above one-third of its water-holding capacity. When calculating evapotranspiration, an adjustment for the atmospheric CO_2_ concentration was made by reducing the reference evapotranspiration by a scaling factor [28]. The value of the scaling factor for 2090 was estimated to be 0.94 for RCP4.5 and 0.88 for RCP8.5 of the baseline value. We used one soil profile for all of the sites, with homogeneous soil properties assumed throughout the top and subsoil layers to enable comparison among sites. The plant-available water at field capacity was assumed to be 270 mm in the entire profile (a depth of 1.3 m). We used a single free-draining soil with good water-holding properties and a relatively deep profile, allowing us to easily perform between-site comparisons of the climate signal.

### 4.3. Defining adverse weather events

To describe the major adverse conditions for wheat production, we used the following set of 11 indicators: indicators of frost damage, water logging, lodging, heat stress, drought stress and adverse conditions during sowing and harvest, with a more detailed description being available in the electronic supplementary material, appendix table S1. To provide a measure of the potential productivity of a given site, we used the sum of the EfGr. We calculated the cumulative global radiation for days with a daily mean air temperature above 5°C, daily minimum air temperature above 0°C, no snow cover and actual-to-reference evapotranspiration ratio above 0.4. To define subregions and assign appropriate weights, Thiesen polygons (figure 1*g*) were used to assign areas represented by each station. Then, the area of arable land in each polygon was estimated using data by Monfreda *et al.* [14]. The weight of each polygon in total wheat production (figure 1*h*) was calculated based on gridded information on wheat acreage [14] combined with FAOSTAT [29] and EUROSTAT 1999–2013 [30] mean yield data (on national or regional level).

Detailed information on the methods and thresholds used as well as associated references are available in the electronic supplementary material, appendix I.

### 4.4. AgriClim software

The agroclimatic parameters listed in the electronic supplementary material, appendix table S1 were calculated with the use of a software package, AgriClim [31]. The software uses daily maximum and minimum temperatures, daily sum of solar irradiation and rainfall, mean daily wind speed and mean daily relative air humidity. For all of the ETr and ETa calculations, the winter wheat canopy was considered using the single crop coefficient (Kc) approach defined by Allen *et al.* [32]. Compared to the original methodology [32], AgriClim accounts for the degree-day driven change of Kc, crop height and rooting depth, which is based on results of Olesen *et al.* [27]. The model also distinguishes between solid and liquid precipitation [33] and the effect of snowmelt on soil water content. An evaluation of the soil moisture routine has been presented, for example, by Hlavinka *et al.* [34].

## Supplementary Material

Supplementary Material - Extended methodology

## Data accessibility

Climate data and weather generator tools used to prepare the paper can be found at LARSWG (http://www.rothamsted.ac.uk/mas-models/larswg.php) and the CMIP5 ensemble distribution site (http://pcmdi9.llnl.gov/).
