# Supplementary material for: Adaptation options for wheat in Europe will be limited by increased adverse weather events under climate change
Source: J R Soc Interface. 2015 Nov 6;12(112):20150721. doi: 10.1098/rsif.2015.0721 (PMC4685845; doi:10.1098/rsif.2015.0721)
Supplement: Supplementary Material - Extended methodology [file rsif20150721supp1.doc]

**Supplementary Material**

***Extended methodology***

The simulation was run for 379 European sites selected as representative from approximately 12,000 25-km grid-cells of the ELPIS datasets of site parameters integrated in to the LARS-WG weather generator (25) using a daily time step (Fig. 1g). These sites cover 36 European countries, covering the current major wheat-producing regions of the EU (Fig. 1h) as well as areas where wheat might be grown in the future (e.g., Elsgaard et al. (35)). The daily reference evapotranspiration (ET_r_) was estimated using the Pristley and Taylor ([3](#_ENREF_1)6) method, as reliable input data for the calculation of the aerodynamic term (wind speed and relative humidity) of the Penman-Monteith equation were not available in the weather database. The actual (ET_a_) evapotranspiration and soil moisture content were then calculated using the approach described by Allen et al*.* (32) and including modifications in the soil water flow and snow cover influence that were validated by Hlavinka et al*.* ([3](#_ENREF_3)4). The crop coefficient values varied with the phenological stages between 0.4 for the bare soil conditions after sowing to 1.1 around heading and 0.8 at maturity. When calculating evapotranspiration, an adjustment for the atmospheric CO_2_ concentration was made (533 ppm for RCP 4.5 and 845 ppm for RCP 8.5) by reducing the reference evapotranspiration by a scaling factor ([2](#_ENREF_31)8) The value of the scaling factor for 2090´s was estimated to be 0.94 (RCP 4.5) and 0.88 (RCP 8.5) of the baseline values. We used one soil profile for all of the sites, with homogeneous soil properties assumed throughout the top and subsoil layers to enable comparison among sites. The plant-available water at field capacity in the top 0.1 m of the soil was assumed to be 20 mm, with 83 mm being stored in the topsoil (up to a 0.4-m depth) and 270 mm in the entire profile (a 1.3-m depth). We used a single free-draining soil with good water-holding properties and a relatively deep profile, allowing us to easily perform between-site comparisons of the climate signal.

The combined probability of occurrence of one or two adverse weather events of those listed in Supplementary Table 1 were calculated using AgriClim, which was designed to allow for a large number of calculations. For all of the ET_a_ calculations, the parameters of wheat (Kc, plant height) were used as the reference crop surface using the parameters derived by Allen et al., (32) which changed according to the crop phenology stage. The duration of the phenological phases was calculated as described by Olesen et al. ([2](#_ENREF_6)7) using the accumulated degree days (°Cd) above the base temperature combined with the day-length response for the period from emergence to anthesis. Supplementary Table 2 lists the parameters that were used for early-, medium- and late-maturing cultivars and photoperiodic sensitivity. The study was focused on studying the exposure of wheat crop to adverse weather conditions and therefore, primary role of the phenological model was to specify exposure windows for particular adverse weather events considered (e.g. time of sowing, anthesis or maturity). Therefore, we were not primarily concerned if winter wheat (i.e. the crop requiring vernalization) or spring wheat types will/should be grown at the site. We also did not include the not-fulfilling vernalization requirements as adverse weather event. The principal argument was that even under baseline climate at some locations farmers can adapt quickly to changes in vernalization requirements. We thus assumed that these cultivars represent both winter and spring wheat, as some locations (mainly in southern Europe) present constraints on the vernalisation requirements. The sowing dates were determined automatically as the first day after the mean air temperature dropped below 13°C for more than five subsequent days. The earliest sowing date allowed was set as August 20th and the latest as December 30th. The minimum required soil moisture for sowing to occur was set at 35% of the maximum plant available water. If the sowing conditions were not met during the prescribed window, then the sowing was performed on December 31^st^.

In order to estimate accumulated probability of whole array of adverse weather events we used 11 indicators of such conditions that were describe in Trnka et al., 2014 (15) and which are summarized in Supplementary Table 1. Their selection was designed to cover the major causes of low yields of wheat across Europe and required not only to represent conditions negatively affecting growth but also those that hamper the ability to sow and harvest the crop at the optimal time. The selection of factors negatively affecting crop yield relied on the analysis of Olesen et al*.* (22) and included the following: indicators of frost damage, water logging, lodging, heat stress, drought stress and adverse conditions during sowing and harvest. The defined thresholds for the indices were based on a combination of literature and expert judgement (21) and are generally set at levels that are likely to cause a severe yield reduction or even crop failure. The study was designed to specifically address the probability of the future occurrence of the most notable adverse and extreme events with the most significant consequences on the yield reduction, as these are commonly not well addressed by process-based crop models (37, 38).

Although the 11 indices focused on the agroclimatic extremes (adverse conditions), we additionally calculated the sum of the effective global radiation ([3](#_ENREF_30)1). This was calculated as the sum of daily global radiation on days when the mean air temperature exceeded of 5°C (without snow cover or frost occurrence) and when plants were not severely limited by lack of water (ETa/ETr > 0.4). Crop growth on a given day is considered to not be significantly limited by water if the daily ratios of ETa to ETr exceed 0.5. For this study, we deliberately chose a lower threshold (0.4) to limit the eventual overestimation of water shortage by the selected indices. It serves in the study as a proxy of a potential productivity summing global radiation only on days with temperature and soil moisture content at levels permitting crop growth and development. It is used in our study to highlight the fact that earlier start of growing season (through enabling earlier sowing and faster development in spring) might come at a price of lower available effective global radiation and result to lower overall productivity (31).

The true impact of adverse weather events in each region will depend not only on the changes in the event frequency but also on the suitability of the given area for the wheat production. Therefore, we calculated the weight of each Thiesen polygon (Fig. 1g) on the basis of wheat area and wheat production. The share of each Thiesen polygon in the total wheat growing area was estimated using data by Monfreda et al. (14) and is depicted at the Fig. 1h. However, there are substantial differences in mean wheat yields among the polygons, which significantly affects the share of each region in the total wheat production. Therefore, the share of each polygon in the total wheat production (Fig. 1h) was calculated based on gridded information on wheat acreage (14) combined with FAOSTAT (29) and EUROSTAT 1999-2013 (30) mean yield data (on national or regional level depending on data availability). The differences in overall results when the wheat area or total wheat production per polygon is considered are clearly demonstrated at Figs. 2 and 3.

*Creating daily weather series for the baseline and future climates*

LARS-WG is a stochastic weather generator that has been widely used in numerous studies on impact assessment of climate change for nearly two decades (39,40). The latest examples include studies by ([41-](#_ENREF_3)50). Recently, its application has been facilitated by integrating a dataset of site parameters for the baseline 1980-2010 European climates (51,52) as well as integration scenarios based on the CMIP3 and CMIP5 ensembles of global climate models (53) and the EU-ENSEMBLE ensemble of regional climate models over Europe (54). This created a unique repository of model parameters, ELPIS, which can be accessed directly within the model interface.

To construct local-scale daily climate scenarios, we used climate projections from a sub-set of 18 global climate models (Supplementary Table 3) from the CMIP5 multi-model ensemble used in the latest IPCC Assessment Report 5 (AR5) (55). These climate projections were developed for new emission scenarios referred as Representative Concentration Pathways (RCPs) (56), that represent a larger set of mitigation scenarios compared with the SRES emission scenarios used in the IPCC AR4 (57). Different RCPs have different targets in terms of radiative forcing at 2100 varying from 2.6 to 8.5 W m^–2^. They all should be considered as plausible, and do not have probabilities attached to them. These RCPs were developed using different Integrated Assessment Models that include economic, demographic, energy, and simple climate components. Two RCPs were integrated in to the LARS-WG, i.e. RCP8.5 which represents rising radiative forcing pathway leading to 8.5 W m^-2^ in 2100**,** and RCP4.5 which represents stabilization without overshoot pathway leading to 4.5 W m^-2^ radiative forcing at stabilization after 2100 (58,59). Corresponding CO2 concentrations (ppm) for RCP4.5 and RCP8.5 were 533 ppm and 845 ppm, respectively.

The CMIP5 multi-model ensemble has more than twice as many models and many more experiments compared with the CMIP3 ensemble. It might be not always practical to use all climate models from the CMIP5 ensemble in a specific impact assessment study. To assist with the selection of GCMs for an impact study, we computed a climatic sensitivity index (CSI) for each model incorporated into LARS-WG. CSI over a region of interest is defined as spatial average (calculated over land only) of differences between values for the future, 2080-2100, for RCP8.5 and values for the CMIP5 “baseline”, 1995-2005. CSI was computed for mean air temperature as an absolute change in temperature (°C), and for precipitation as a relative change of precipitation total (%). Appendix Figrue 3 presents CSIs for Mediterranean Basin (MED) and Northern Europe (NEU) for 18 GCMs. All GCMs predicted an increase in annual precipitation in NEU by up to 25% for MIROC-ESM, and a decrease in annual precipitation in MED by up to -36% for IPSL-CM5A-MR. Changes in mean annual temperature were similar for both regions, NEU and MED, and varied from +3.1 C for INMCM4 to +6.6 C for MIROC-ESM.

In our study we used climate projections from two climate models with very different climate sensitivity, HadGEM2-ES (numbered 9 in Supplementary Table 3 and Appendix Fig. 3) and GISS-E2-R-CC (numbered 8), and two RCPs, RCP4.5 and RCP8.5. HadGEM2-ES projections for 2080-2100 are nearly the hottest and driest (during summer) projections for both north (NEU– latitude 48°-75°N; longitude 10°W-40°E) and south (MED – latitude 30°-48°N; longitude 10°W-40°E) Europe (Appendix Figure 3). GISS-E2-R-CC projections are nearly the coolest projections with changes in precipitation closed to CMIP5 ensemble average (Appendix Figure 3).

Supplementary Table 1. Overview of the 11 agro-climatic indices used in this study.

| **Indicator name**  **[Units]** | **Effect on wheat** | **Indicator description – situation must occur at least once per season to be counted** |
| --- | --- | --- |
| 1. **Frost with no snow** | Symptoms including leaf chlorosis; burning of leaf tips to cut back the plants to replant the fields in the spring | Event is triggered when the T_min_^*^ is equal to or below -20°C for at least one day with no or very limited snow cover^†^ (less than 1 cm of freshly fallen snow) |
| 1. **Late frost** | Its occurrence after loss of the winter-hardiness leads to leaf chlorosis, burning of leaf tips, floret sterility, damage to lower stem and consequently medium to severe yield losses | Event is triggered when the T_min_^*^ is equal to or below -2°C after the start of the following window, determined as the period when the mean air temperature is continuously 10°C (for at least five days) and does not drop below 10°C for more than two days in a row |
| 1. **Extremely wet early season** | Restricts growth and reduces yield through the occurrence of diseases, nitrogen leaching, logging and root anoxia | Event is triggered if the soil moisture is at or above the field capacity for more than 60 days from sowing to anthesis. Days with a mean temperature below 3°C are not counted |
| 1. **Lodging event** | Causes severe reduction of yield and of grain quality, e.g., through increased harvest losses and increased exposure to diseases | Event is triggered if there are at least two days in the season with a daily precipitation on the given day above 40 mm or if the daily precipitation is above 20 mm and the soil moisture on the preceding day is at or above the field capacity; the period from anthesis to five days before maturity is considered |
| 1. **Anthesis heat stress event** | Causes partial or complete sterility of the florets with a severe effect on yield | Event is triggered when the T_max_^‡^ is above +31°C for at least two days during the period ± 5 days around anthesis |
| 1. **Grain-filling extreme heat exposure and accumulation** | Speeds up development and decreases yield until the growth stops, resulting in a substantial yield reduction | Event is triggered when the T_max_^‡^ is above +35°C for at least three days during the period from five days after anthesis to maturity. |
| 1. **Severe drought event sowing – anthesis** | Causes a severe reduction of growth or crop die back | Event is triggered if ET_a_^§^/ET_r_^‖^ is less than 0.15 for at least ten consecutive days between sowing and anthesis; the days with a mean temperature below 3°C are not considered |
| 1. **Severe drought event anthesis-maturity** | Causes a severe reduction of growth or crop die back | Event is triggered if ET_a_/ET_r_ is less than 0.15 for at least ten consecutive days between anthesis and maturity |
| 1. **Severely dry season sowing-maturity** | Causes severe reduction of growth or crop die back | Event is triggered if ET_a_/ET_r_ is less than 0.15 for at least 21 days during the period from sowing to maturity; the days with a mean temperature below 3°C are not considered |
| 1. **Adverse conditions at sowing** | Restricts the ability to use the appropriate sowing window | Event is triggered when there are no more than three days during the sowing window (sowing date ±15 days) with the soil moisture in the top layer below 90% but above 5% and rain on the given day is below 5 mm and not more than 10 mm on the preceding day |
| 1. **Adverse conditions at harvest** | Restricts the ability to harvest at the most appropriate time | Event is triggered when there are fewer than three days during the harvest window (maturity date + five days up to maturity + 25 days) with the soil moisture in the top layer below 85% and rain on the given day is below 0.5 mm and not more than 5 mm on the preceding day |

**Notes:**

^*^The T_min_ minimum daily temperature was measured 2 m above ground; thus, the actual crop temperature might be even lower.

^†^ The snow cover was estimated using a model validated by Trnka et al. (33)

^‡^The T_max_ maximum daily temperature was measured 2 m above ground.

^§^The ET_a_ refers to the actual evapotranspiration calculated for winter wheat assuming a soil water-holding capacity of 0.27 m and a maximum rooting depth of 1.3 m (more details in the text).

^‖^The ET_r_ refers to the same crop surface as (§) but for reference evapotranspiration; the crop parameters were set according to Allen et al. (32)

Supplementary Table 2. Overview of the phenological parameters used in this study.

| **Cultivar Type** | **Sowing - Emergence** | **Emergence - Anthesis** | | | **Anthesis - Maturity** | |
| --- | --- | --- | --- | --- | --- | --- |
|  |  | Calculation | Parameters | Calculation | | Parameters |
| **Early** | The time from sowing to emergence was calculated with T_b_ = 0°C and T_sum_ = 125°C | 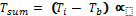  Where  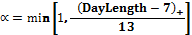 | T_b_ = 4°C and T_sum_ = 380°C | 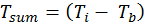 | | T_b_ = 6°C and T_sum_ = 280°C |
|  |  |  |  |  |  |  |
| **Medium** |  |  | T_b_ = 4°C and T_sum_ = 460°C |  |  | T_b_ = 6°C and T_sum_ = 420°C |
|  |  |  |  |  |  |  |
| **Late** |  |  | T_b_ = 4°C and T_sum_ = 540°C |  |  | T_b_ = 6°C and T_sum_ = 560°C |

Supplementary Table 3. Global climate models from the CMIP5 ensemble incorporated in the LARS-WG weather generator. Scenarios are based on RCP4.5 and RCP8.5 Representative Concentration Pathways.

| # | Research Centre | Country | Global climate model | Grid resolution | Reference |
| --- | --- | --- | --- | --- | --- |
| 1 | The Centre for Australian Weather and Climate Research | Australia | ACCESS1-3 | 1.25° x 1.88° | (60) |
| 2 | Beijing Climate Center | China | BCC-CSM1.1 | 2.77° x 2.81° | (61) |
| 3 | Canadian Centre for Climate Modelling and Analysis | Canada | CanESM2, | 2.77° x 2.81° | (62) |
| 4 | Centro Euro-Mediterraneo sui Cambiamenti Climatici | Italy | CMCC-CM | 0.74° x 0.75° | (63) |
| 5 | CNRM-GAME & Cerfacs | France | CNRM-CM5 | 1.40° x 1.40° | (64) |
| 6 | Australia's Commonwealth Scientific and Industrial Research Organisation | Australia | CSIRO-MK36 | 1.85° x 1.88° | (65) |
| 7 | EC-Earth consortium | Europe | EC-EARTH | 1.125° x 1.125° | ([6](#_ENREF_31)6) |
| 8 | Goddard Institute for Space Studies | USA | GISS-E2-R-CC | 2.00° x 2.50° | ([6](#_ENREF_32)7) |
| 9 | UK Meteorological Office | UK | HadGEM2-ES | 1.25° x 1.88° | ([68-7](#_ENREF_33)0) |
| 10 | Institute for Numerical Mathematics | Russia | INM-CM4 | 1.50° x 20° | (71,72) |
| 11 | Institute Pierre Simon Laplace | France | IPSL-CM5A-MR | 1.27° x 2.50° | ([7](#_ENREF_38)3) |
| 12 | University of Tokyo, National Institute for Envir. Studies, Japan Agency for Marine-Earth Science & Technology | Japan | MIROC5 | 1.39° x 1.41° | ([74,7](#_ENREF_39)5) |
| 13 | University of Tokyo, National Institute for Envir. Studies, Japan Agency for Marine-Earth Science & Technology | Japan | MIROC-ESM | 2.77° x 2.81° | ([7](#_ENREF_39)6) |
| 14 | Max-Planck Institute for Meteorology | Germany | MPI-ESM-MR | 1.85° x 1.88° | (77,78) |
| 15 | Meteorological Research Institute | Japan | MRI-CGCM3 | 1.11° x 1.13° | (79) |
| 16 | National Centre for Atmospheric Research | USA | NCAR-CCSM4 | 0.94° x 1.25° | (80,81) |
| 17 | National Centre for Atmospheric Research | USA | NCAR-CESM1-CAM5 | 0.94° x 1.25° | (80) |
| 18 | Norwegian Climate Centre | Norway | NorESM1-M | 1.90° x 2.50° | (82,83) |

***Appendix – Figures***


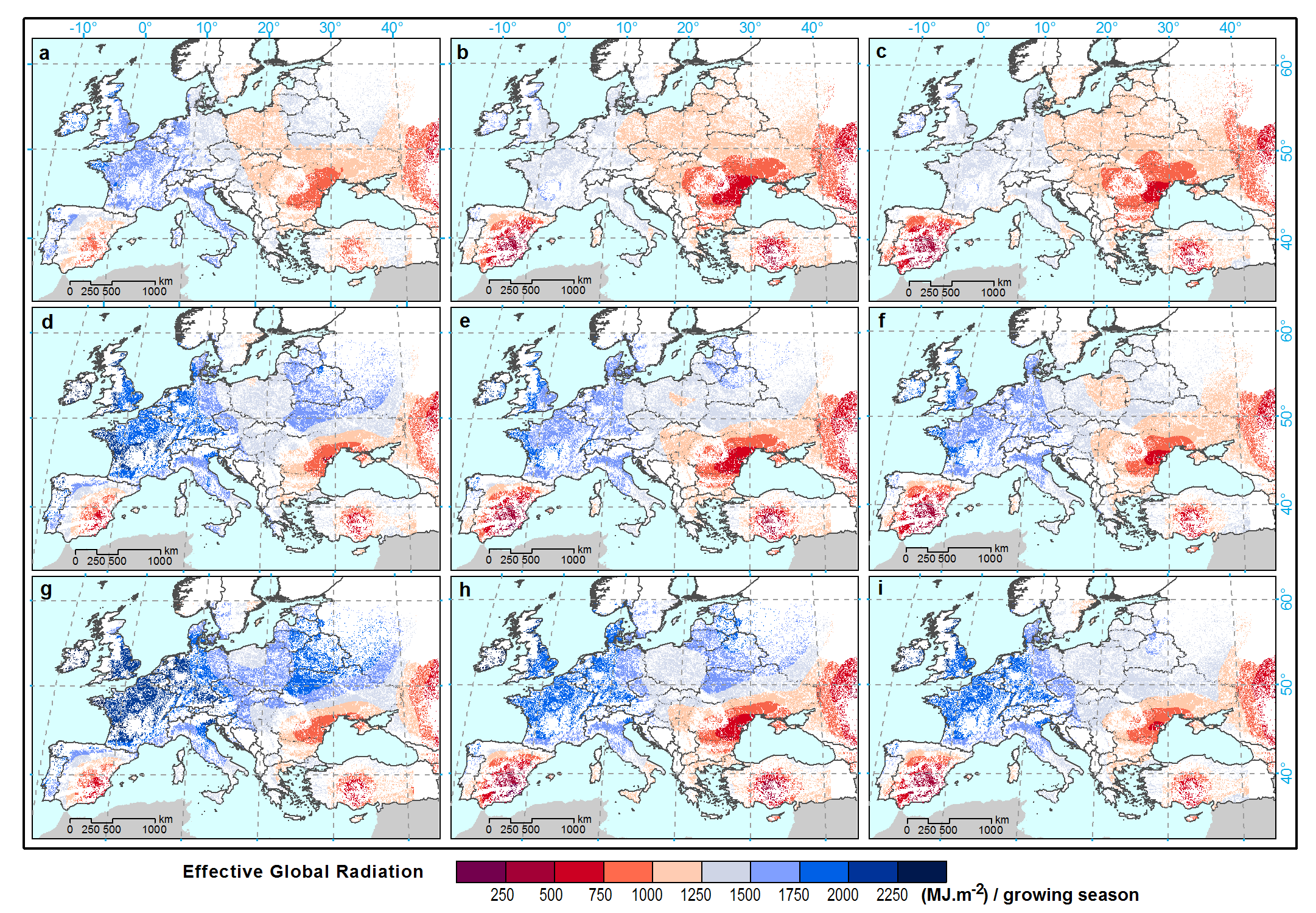


Appendix Figure 1: Mean effective global radiation for early (a-c), medium (d-f) and late (g-i) cultivars during the period from sowing to maturity. The baseline is represented by the most left column. The medium column represents GISS-RCP8.5 and most right column HadGEM-RCP8.5 climate scenarios for 2081-2100. The map is based on the values of 379 stations from which the mean effective global radiation was interpolated through co-krigging accounting for the altitude. Only values for arable land (14) are shown.


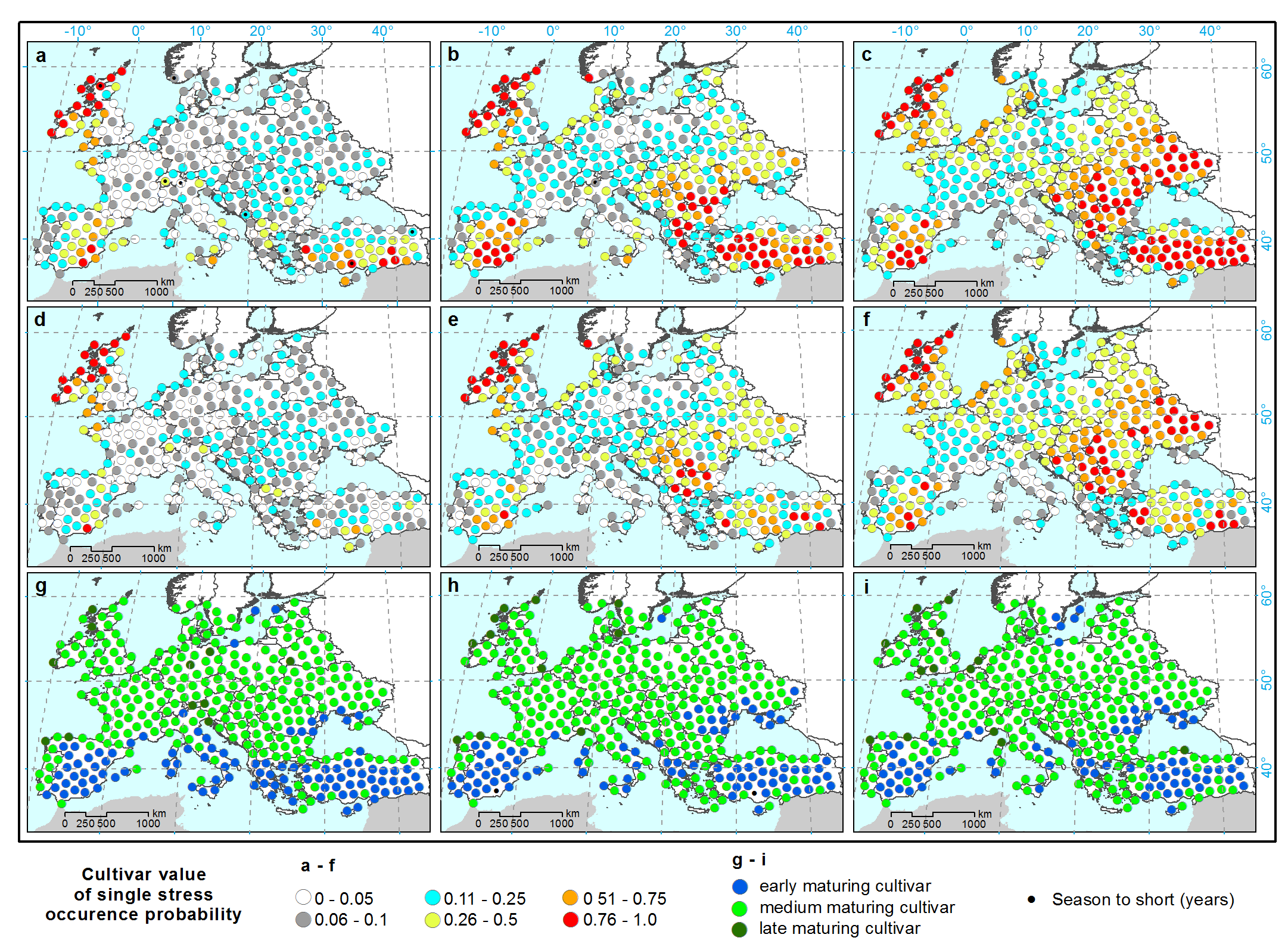


Appendix Figure 2: Combined probability of single adverse event occurrence for baseline (a), GISS-RCP8.5 (b) and HadGEM-RCP8.5 (c) using medium maturing cultivar. Figures (d)-(f) indicate the same combined probability after the cultivar optimization i.e. each site is represented by the cultivar with lowest adverse event probability (without decreasing effective global radiation value by more than 5% compared to baseline). The g-i shows, which kind of cultivar was selected based on the optimized cultivar selection used for maps d-f. The black point indicates conditions unsuitable for any of the cultivars.


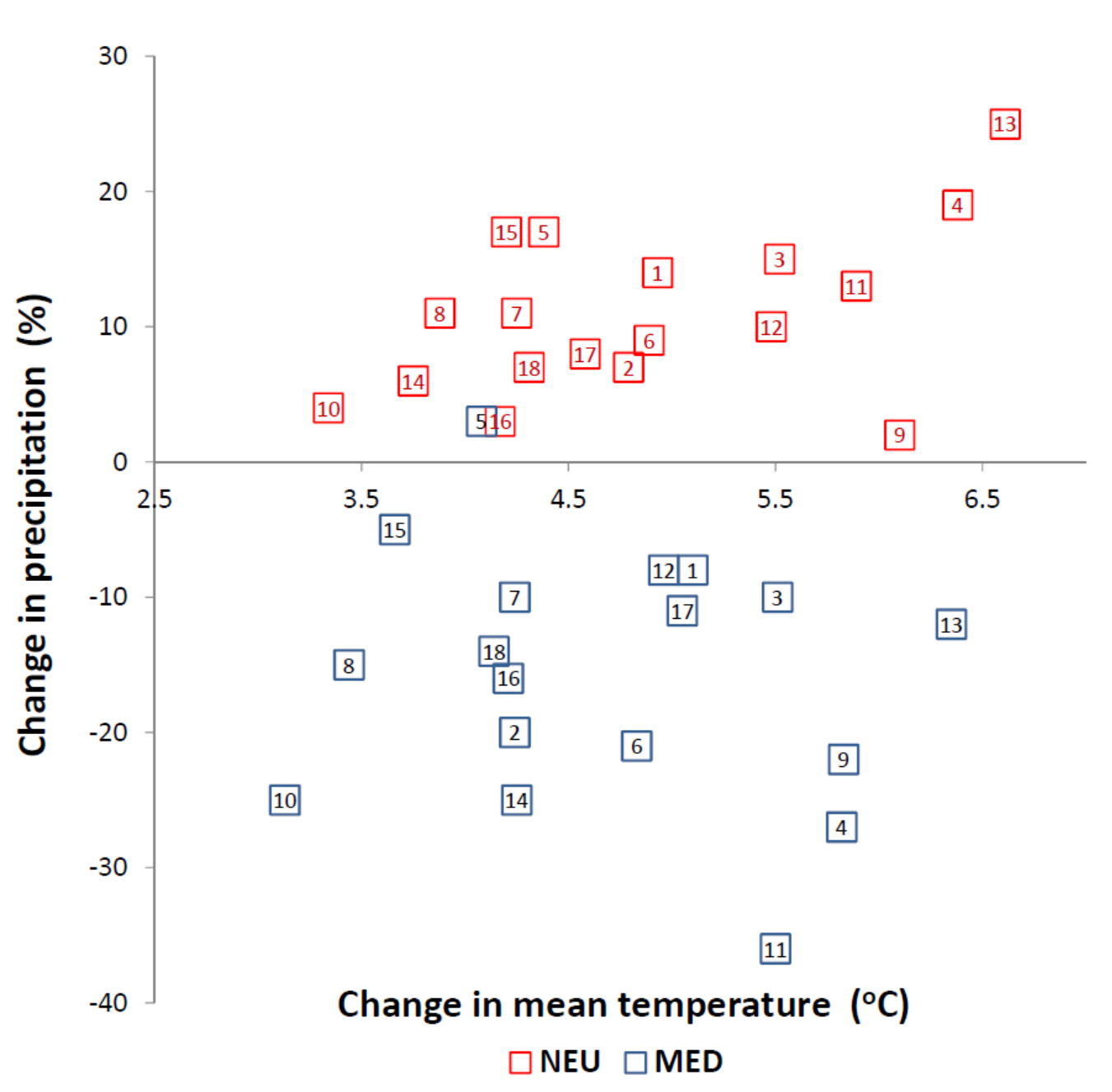


Appendix Figure 3: Absolute changes in mean annual temperature (^o^C) and relative changes in annual mean precipitation calculated over Northern Europe (NEU, red) and Mediterranean Basin (MED, blue) regions between future 2080-2100 and GCM baseline 1995-2005 for 18 GCMs from the CMIP5 ensembles. GCMs descriptions are given in Supplementary Table 3 with [8] standing for the GISS model and [9] for the HadGEM model. Values are calculated for land grid-cells from the 1-degree land mask.


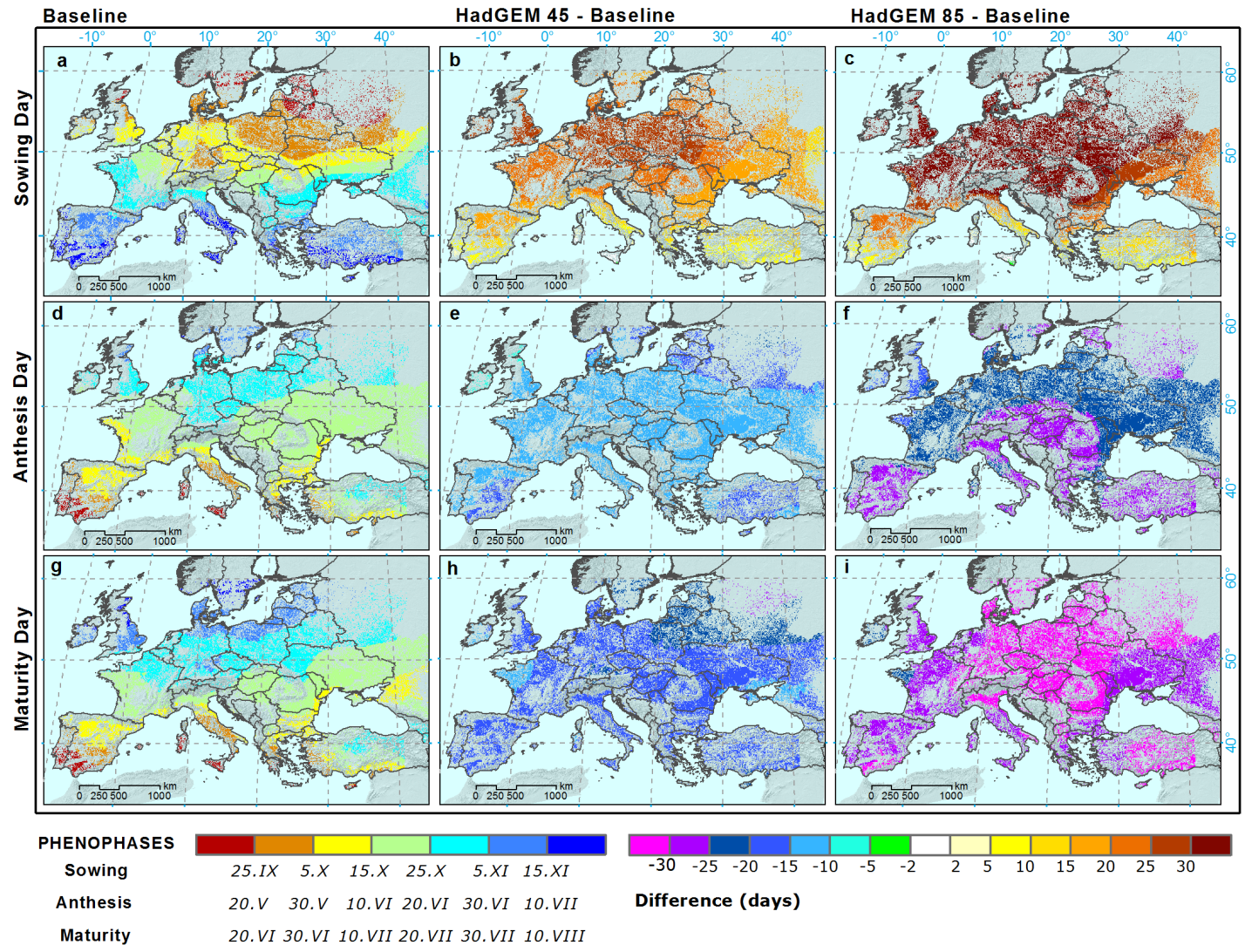


Appendix Figure 4: Mean sowing (a), anthesis (d) and maturity (g) dates for the baseline conditions and the mean shift of these dates for HadGEM RCP4.5 (b,e,h) and HadGEM-RCP8.5 (c,f,i) for sowing (b,c), anthesis (e,f) and maturity (h,i) using medium cultivar. Only values for arable land (14) are shown.

***Supplementary References***

1. Elsgaard L, et al. (2012) Shifts in comparative advantages for maize, oat and wheat cropping under climate change in Europe. *Food Addit Contam A* 29: 1514–1526.
